# Editorial: Competency frameworks in health professions education

**DOI:** 10.3389/fmed.2022.1034729

**Published:** 2022-10-06

**Authors:** Claire Palermo, H. Thomas Aretz, Eric S. Holmboe

**Affiliations:** ^1^Faculty of Medicine, Nursing and Health Sciences, Monash University, Clayton, VIC, Australia; ^2^Mass General Brigham, Global Advisory, Harvard Medical School, Boston, MA, United States; ^3^Accreditation Council for Graduate Medical Education, Chicago, IL, United States

**Keywords:** competency based education, competency based assessment, curriculum, health workforce, workforce capabilities

Competency-based health professions education is widely implemented as the framework for preparing health professionals for practice. Competency-based health professions education is inclusive of competency or practice standards that articulate the knowledge, skills, attitudes, and capabilities of a profession; the curriculum and assessment that supports achievement and measurement against these standards, and accreditation policies and procedures (1, 2). Competency-based assessment in the workplace is a critical part of competency-based education. Recent advances in assessment include programmatic approaches (3), trustworthy professional activities (4), and milestones (2) to support shared understanding of competence. While there are justifiable concerns surrounding a competency-based approach, for example, the focus on individual rather than the collective competence of interdisciplinary teams who provide healthcare (5), this framework remains central to the preparation of health professionals.

This Research Topic, which is part of *Frontiers in Medicine Health Professions Education*, presents a collection of current research on competency-based health professions education. The contributors provide new knowledge and recommendations for a range of issues related to competency-based education. Three literature reviews synthesize evidence for developing competency frameworks (Batt et al.; Lepre et al.; Murray et al.). Drawing from the literature, Batt et al. provide a six-step framework as guidance on best practices in the development of competency frameworks. Together with the two other reviews (Lepre et al.; Murray et al.), the manuscripts provide guidance for those undertaking the development or revision of competency frameworks showcasing the diversity of methods used (Batt et al.; Murray et al.). The reviews highlight the limited diversity of stakeholders involved in competency framework development (Lepre et al.) especially patients or the communities health professionals serve (Murray et al.). This is a critical finding given that ensuring patient and community health involvement results in an improvement in service delivery and assures needs are addressed (6). Moving forward, ensuring patient and public involvement in competency frameworks at a minimum, through co-production is critical for improving educational programs to produce better health outcomes (7).

The studies in this section present research on new approaches to competency development, such as Q-methodology (Chang et al.) and document analysis (Allen and Palermo). Of particular interest is the use of document analysis, which potentially helps those in resource poor environments to ensure standards remain up-to-date. This approach may manage another criticism of the competency movement, which suggests that by the time standards are already written they are out of date and cannot reflect the complex nature of healthcare. As health care professionals, educators are challenged to look beyond these standards. They need to consider current trends in health services, systems, and community health needs and adapt the curriculum in time, working proactively to push standards to evolve and keep pace with changes in biological, clinical, educational, and system science evidence. This requires a growth mindset and leadership at all levels that embraces trial and learning through experimentation (8).

This collection also presents research on innovative methods for building the competence of the existing workforce (Valentim et al.) together with advanced practice competency frameworks to address the emerging health needs of populations (Meilianti et al.). Ensuring health professionals are prepared to meet the emerging health needs of populations is arguably the greatest challenge for competency-based education but is well aligned with the core philosophy of competency-based education (9). Competency frameworks provide the architecture to prepare the health workforce for practice and ensure that those in the workforce remain able to undertake the work the community and health systems requires.

The current pace of health professions education systems to adapt to change is inadequate to ensure health workforces meet the current and future needs of the communities in which they serve. This is further compounded by the complex process and time it takes to translate health professions education research into education practice (10). Education systems across the globe are still struggling to fully implement competency-based education. The reasons are many and vary depending on local contexts, such as rigid and unmalleable curricular structures, insufficient or poorly designed learning opportunities, dysfunctional clinical learning environments, and local or regional regulatory practices, such as accreditation, that are prohibitive of rapid change that threatens or is perceived to threaten the status quo. Compounded by an over-loaded curriculum, there is a reluctance to introduce new content and limited disciplines that have adopted concept-based approaches (11). There is an opportunity and need to more effectively and appropriately involve the end-users of health care and systems that health professions provide, enabling professionals to better meet health needs (12). Given these complexities, responsibilities lie with health profession educators to lead innovation and change.

This Research Topic provides a handbook of guidance for those embarking on standards revisions or development. What would it take to ensure the system is more adaptive and responsive? What does competency-based health profession education look like when it is developed by patients, their families, and communities or partnership with students? What does competency-based health profession education look like when it is collective and interprofessional? Why do we need to wait until accreditation systems push change in curricula when we know what is essential for producing work-ready health profession graduates?

Implementing any major systems change is difficult and the process of fully realizing competency-based education will be no different (13). The good news is that we currently possess the educational tools for competency-based education—we need to work on how best to use them in our educational programs (13). Two important fields of health profession training that educators should embrace are translational and implementation science. These disciplines provide helpful guidance on *how* to effect change and implement new procedures and processes (14, 15). This collection provides some excellent examples and guidance, such as including the affected stakeholders in the change. The use of co-production principles can help to accelerate the adoption of competency-based education more effectively (12). Finally, many challenges are similar across countries and regions. We recommend that regulatory bodies, such as accreditation, certification, and licensure, collaborate to seek ways they can collectively create more space and support for innovation. We encourage educators to embrace innovation and change so that future health professionals can better meet health needs.

## Author contributions

CP conceptualized the editorial with input from EH and HA. CP drafted the editorial and EH and HA provided critical review, feedback, and approved final version of manuscript. All authors contributed to the article and approved the submitted version.

## Conflict of interest

The authors declare that the research was conducted in the absence of any commercial or financial relationships that could be construed as a potential conflict of interest.

## Publisher's note

All claims expressed in this article are solely those of the authors and do not necessarily represent those of their affiliated organizations, or those of the publisher, the editors and the reviewers. Any product that may be evaluated in this article, or claim that may be made by its manufacturer, is not guaranteed or endorsed by the publisher.
